# Supplementary material for: Exceptional Evolution of a Squamous Odontogenic Tumor in the Jaw: Molecular Approach
Source: Int J Mol Sci. 2024 Sep 2;25(17):9547. doi: 10.3390/ijms25179547 (PMC11395408; doi:10.3390/ijms25179547)
Supplement: Supplementary file 1 [file ijms-25-09547-s001.zip › ijms-3143882-supplementary.pdf]

Supplementary Figure 1

A: B-Catenin

Initial lesion | Recurrent lesion

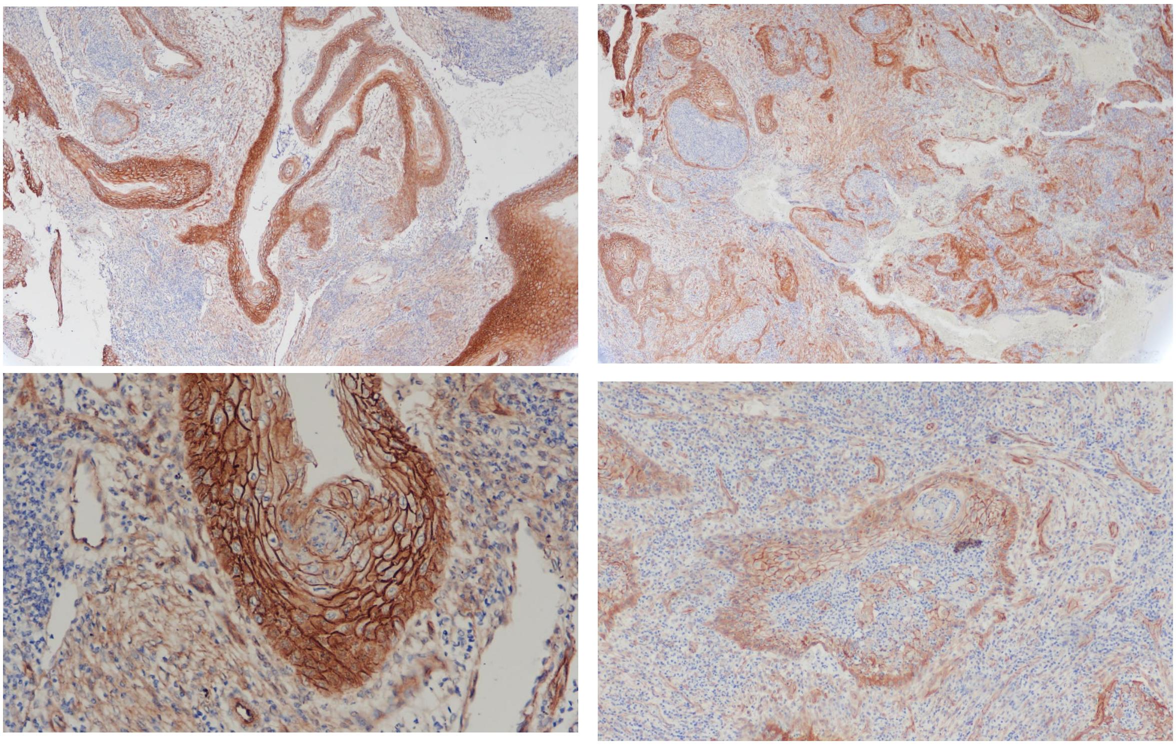

B: Ki67

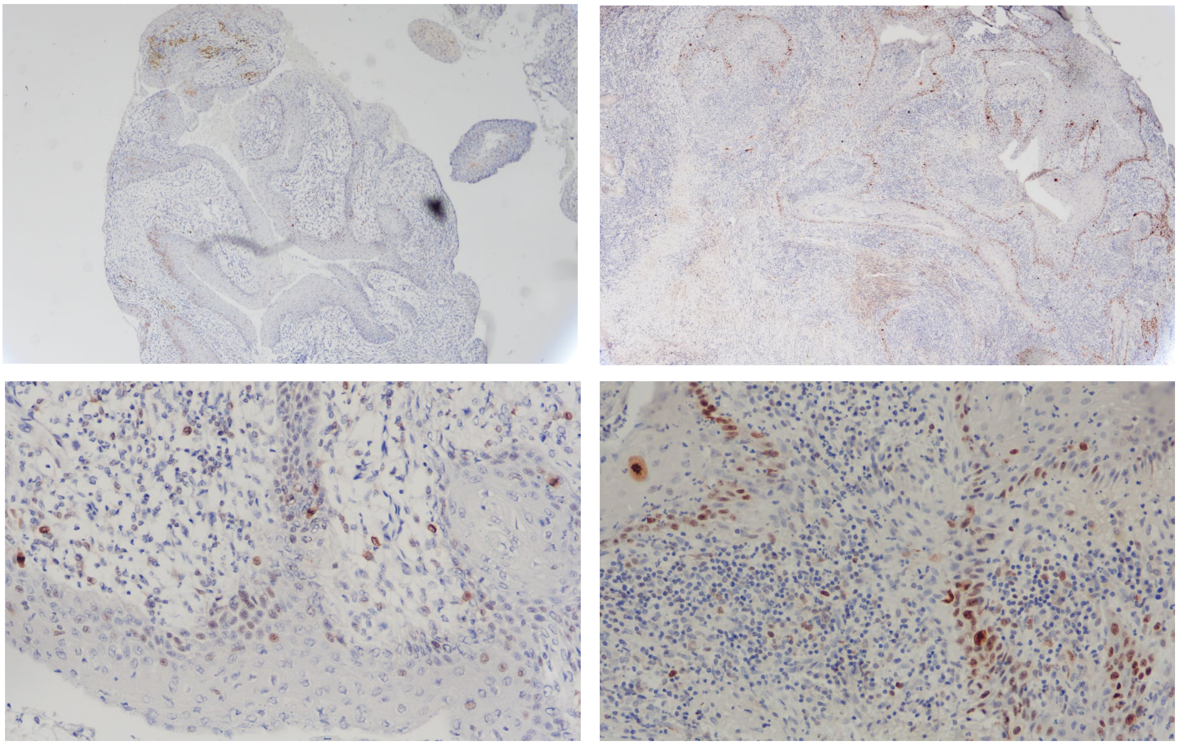

C: P53

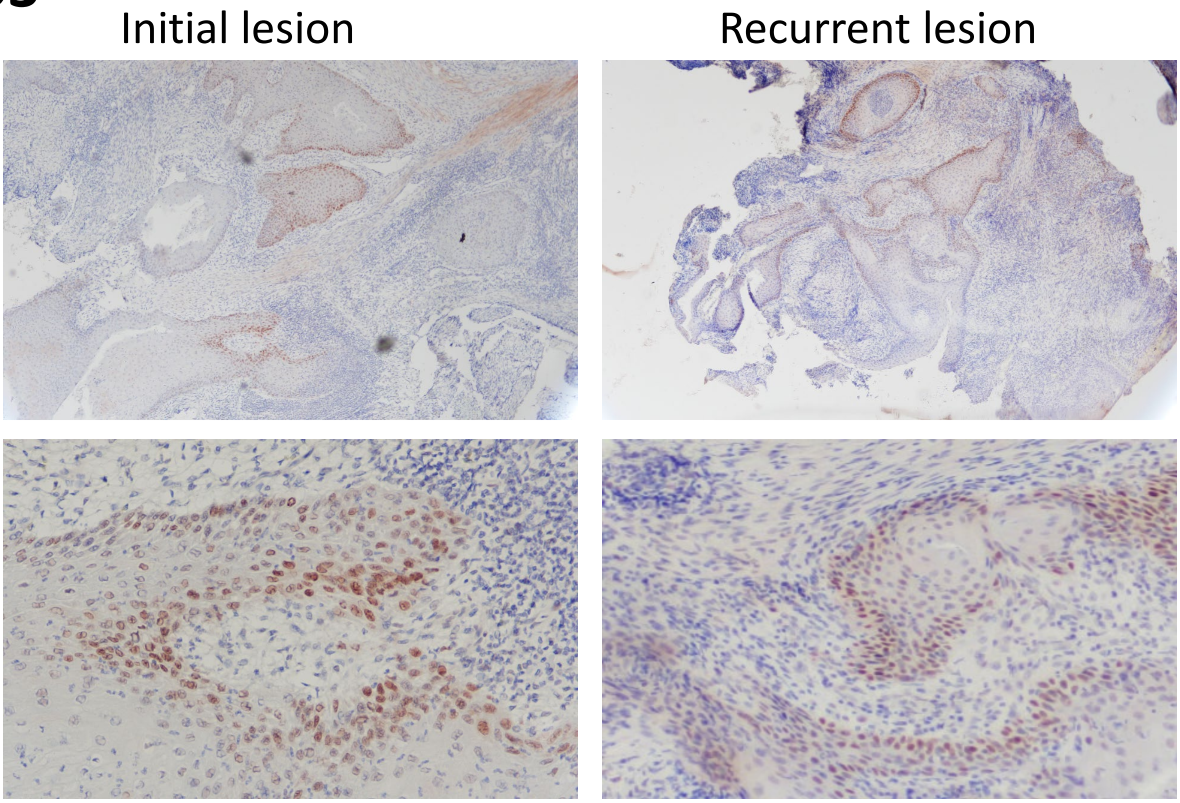

Supplementary Figure 1. Immunohistochemistry expression of β-catenin (A), Ki67 (B) and P53 (C) in the initial and recurrent lesion. Membrane β-catenin delocalization (A) as well as an increase in the expression of Ki65 (B) and P53 (C) is observed in the recurrent lesion.
